# Mortality risk of femoropopliteal paclitaxel-coated devices remains inconclusive

**DOI:** 10.1007/s12928-019-00573-1

**Published:** 2019-02-08

**Authors:** Mitsuyoshi Takahara, Osamu Iida, Yoshimitsu Soga, Daizo Kawasaki, Masahiko Fujihara

**Affiliations:** 1 Department of Diabetes Care Medicine, Osaka University Graduate School of Medicine, Suita, Japan; 2 Cardiovascular Center, Kansai Rosai Hospital, 3-1-69 Inabaso, Amagasaki, Hyogo 660-8511 Japan; 3 Department of Cardiology, Kokura Memorial Hospital, Kitakyushu, Japan; 4 Cardiovascular Center, Morinomiya Hospital, Osaka, Japan; 5 Department of Cardiology, Kishiwada Tokushukai Hospital, Kishiwata, Japan

We read with great interest the recent manuscript by Katsanos and colleagues in Journal of the American Heart Association [1]. They performed a meta-analysis and concluded that there was an increased risk of death following application of paclitaxel balloons and stents in the femoropopliteal artery of the lower limbs. Although they carefully conducted statistical analysis, it would be better to mention some study limitations derived from the incompleteness of the original data they obtained.

First, in their meta-analysis, the mortality risk was assessed based on the risk ratio (RR) calculated from the number of observed events and that of total subjects. For example, in the ZILVER-PTX trial in Fig. 3, the number of observed events and that of total subjects were 42 and 297 in the Paclitaxel group, and 12 and 177 in the Control group. The risk ratio was then calculated to be (42/297)/(12/177) = 2.09. However, in the calculation, censored cases were ignored, which would cause an inaccurate estimation of the mortality risk. Let us go back to the original article [2]. The original article showed that 37 deaths were observed in the primary drug-eluting stent (DES) group (*n *= 236), and 17 deaths in the percutaneous transluminal angioplasty (PTA) group (*n *= 238). According to the aforementioned manner, the risk ratio can be calculated to (37/236)/(17/238) = 2.19, apparently suggesting a higher mortality risk in the primary DES group versus the PTA group. However, this does not correctly reflect the study result. In the trial, as clearly described in the article [2], “the 5-year all-cause mortality rate was 13.6% (10.2% for the primary DES group and 16.9% for the PTA group, *P* = 0.03)”. This would be a good example telling us that a careful interpretation would be required when the mortality risk was assessed simply from the number of observed events and that of total subjects.

Second, it should be noted that most original trials were conducted to primarily assess patency rather than mortality risk. In such randomized controlled trials, baseline characteristics were often similar regarding to restenosis risk, but it remained uncertain whether the allocated groups had similar baseline characteristics regarding the mortality risk. In addition, we must remember that, for example, in the aforementioned ZILVER-PTX trial [2], some subjects originally allocated to the PTA group finally underwent bail-out DES implantation. There might be some critical difference in baseline characteristics between the bail-out DES subgroup and the successful PTA subgroup. Even if baseline characteristics were similar between the primary DES group and the PTA group, they might differ between the DES-implanted group (the primary DES group plus the bail-out DES subgroup) and the successful PTA subgroup. An apparently increased risk of mortality in the paclitaxel-coated stent/balloon group might come from such difference in baseline characteristics between the groups.

The statistical results demonstrated in the article [1] might be statistically correct and might show us something statistically important. However, great caution is needed when interpreting the results.
